# Maternal Choline Supplementation and High-Fat Feeding Interact to Influence DNA Methylation in Offspring in a Time-Specific Manner

**DOI:** 10.3389/fnut.2022.841787

**Published:** 2022-01-28

**Authors:** Hunter W. Korsmo, Bhoomi Dave, Steven Trasino, Anjana Saxena, Jia Liu, Jorge Matias Caviglia, Kaydine Edwards, Moshe Dembitzer, Shameera Sheeraz, Sarah Khaldi, Xinyin Jiang

**Affiliations:** ^1^Department of Biochemistry, The Graduate Center, City University of New York (CUNY), New York, NY, United States; ^2^Department of Health and Nutrition Sciences, Brooklyn College of the City University of New York, Brooklyn, NY, United States; ^3^School of Urban Public Health, Hunter College of the CUNY, New York, NY, United States; ^4^Department of Biology, Brooklyn College of the City University of New York, Brooklyn, NY, United States; ^5^Advanced Science Research Center at the Graduate Center of the CUNY, New York, NY, United States

**Keywords:** choline, high-fat, maternal obesity, DNA methylation, fetal programming

## Abstract

Maternal methyl donor supplementation during pregnancy has demonstrated lasting influence on offspring DNA methylation. However, it is unknown whether an adverse postnatal environment, such as high-fat (HF) feeding, overrides the influence of prenatal methyl donor supplementation on offspring epigenome. In this study, we examined whether maternal supplementation of choline (CS), a methyl donor, interacts with prenatal and postnatal HF feeding to alter global and site-specific DNA methylation in offspring. We fed wild-type C57BL/6J mouse dams a HF diet with or without CS throughout gestation. After weaning, the offspring were exposed to HF feeding for 6 weeks resembling a continued obesogenic environment. Our results suggest that maternal CS under the HF condition (HFCS) increased global DNA methylation and DNA methyltransferase 1 (*Dnmt1*) expression in both fetal liver and brain. However, during the postnatal period, HFCS offspring demonstrated lower global DNA methylation and *Dnmt1* expression was unaltered in both the liver and visceral adipose tissue. Site-specific DNA methylation analysis during both fetal and postnatal periods demonstrated that HFCS offspring had higher methylation of CpGs in the promoter of *Srebf1*, a key mediator of *de novo* lipogenesis. In conclusion, the influence of maternal CS on offspring DNA methylation is specific to HF feeding status during prenatal and postnatal periods. Without continued CS during the postnatal period, global DNA methylation enhanced by prenatal CS in the offspring was overridden by postnatal HF feeding.

## Introduction

Epigenetic marks are heritable modifications on the chromosomes that are pivotal in controlling gene expression during normal physiological development and in response to pathological conditions. DNA methylation is one of the major mechanisms that direct organismic epigenome. Choline, a semi-essential nutrient, participates in phospholipid and one carbon metabolism (OCM) (1). When choline is oxidized to betaine, its labile methyl group can be used for homocysteine remethylation to methionine. Thereafter, methionine is converted to the universal methyl donor *S*-adenosylmethionine (SAM). Therefore, choline-derived methyl group is used for various methylation reactions, including those that affect epigenetic modifications.

A series of landmark studies using the viable yellow agouti obese mouse model demonstrated that supplementing the maternal diet with one carbon nutrients including choline, betaine, folate, and vitamin B_12_ increased DNA methylation of the intracisternal A particle (IAP) retrotransposon at the *agouti* locus and other genomic loci in the offspring, thereby preventing altered fur coat color and obesity in postnatal life of offspring (2–4). Modifying the availability of choline alone during pregnancy has also been shown to elicit DNA methylation changes in offspring tissues. Choline supplementation (CS) during pregnancy increased global DNA methylation of both fetal livers in the toxic milk (tx-j) mouse model (5) and placental tissue in the non-Swiss Albino mice (6). Women who received higher choline intake in the 3rd trimester of pregnancy, similarly had increased global DNA methylation of the placenta (7). Paradoxically, in the same human study, higher maternal choline intake led to lower DNA methylation of genes encoding corticotropin releasing hormone (*CRH*) and glucocorticoid receptor (*NR3C1*) in fetal cord blood, demonstrating an inverse correlation of maternal choline intake and fetal DNA methylation (7). Conversely, gestational choline deficiency in rat, led to a higher DNA methylation of the insulin-like growth factor 2 (*Igf2*) gene in the fetal brain, possibly due to lower methylation of promoter CpG island thereby increased expression of *Dnmt1*, the enzyme mediating DNA methylation (8). These observations suggest an intricate relationship between maternal choline intake and offspring epigenetics rather than simple substrate availability. In addition, most of the existing studies present a snapshot of how maternal choline influences offspring epigenetics at one single time point during the fetal or neonatal period. Therefore, large gaps of knowledge on the dynamic changes in the relationship between maternal CS and offspring epigenetics that extend beyond the fetal period into postnatal life remain.

It is also unclear how other prenatal and postnatal nutrition exposures may interact with maternal CS in determining the epigenome of offspring. Maternal obesity has become a major public health concern that predisposes offspring to increased risk of cardiometabolic diseases. Moreover, when the offspring are also exposed to a postnatal obesogenic environment, their risks of adverse metabolic outcomes such as glucose intolerance and hepatic steatosis are further exacerbated (9–14). We have previously found that maternal CS reduced the negative effects of maternal HF-induced obesity and gestational diabetes, normalizing fetal growth, preventing excess in fetal adiposity, and reducing glucose intolerance of offspring when they were exposed to an obesogenic diet after birth (15–17). It remains to be determined whether these observed phenotypic benefits were mediated in part by the influence of maternal CS on epigenetic programming.

The current view on the effects of HF feeding on pre- and post- natal DNA methylation is complex and contradictory. Peng et al. reported that maternal HF feeding led to global hypermethylation of the offspring liver, with disturbances in OCM metabolites (18). Vucetic et al., however, demonstrated that maternal HF feeding reduced both global DNA and μ-opioid receptor (MOR) promoter methylation in the mouse brain, leading to higher MOR expression and preference for HF and high-sugar palatable foods (19). Maternal methyl donor supplementation reversed the hypomethylating effect of HF feeding in the offspring (20). Overall, the impact of maternal HF on offspring methylation seems to be tissue-specific and depends, in part, on one carbon nutrient status. Intriguingly, postnatal HF feeding seems to override most of the effect of prenatal HF on the epigenome as demonstrated by another study (21). It is therefore important to determine the dynamic changes in DNA methylation of offspring in response to prenatal and postnatal obesogenic environments and whether maternal CS can establish and maintain DNA methylation against the epigenetic influence of prenatal and postnatal HF feeding.

In this study we aimed to determine the time-specific effect of maternal CS on epigenetic changes in offspring exposed to prenatal and postnatal obesogenic environments. We hypothesized that epigenetic marks established by CS during the prenatal period would be eliminated by a postnatal obesogenic environment. We examined global and site-specific DNA methylation of offspring exposed to maternal CS and HF feeding both during the fetal period and in early adulthood.

## Materials and Methods

### Animals and Diets

The study protocol was approved by the Institutional Animal Care and Use Committee (IACUC) at Brooklyn College. Six-week old C57BL/6J mice were obtained from the Jackson Laboratory. They were housed at 22°C, humidity 40–60%, and 12-h light/dark cycle with regular bedding and enrichment. The mice were fed a regular lab diet (Laboratory Rodent Diet 5012, LabDiet, St. Louis, MO, USA) for 2 weeks during acclimation. Thereafter, female mice were fed one of the following diets *ad libitum*: the normal-fat control (NFCO) group received a normal-fat (NF) diet (D12450J, Research Diets, New Brunswick, NJ, USA) containing 10% kcal from fat and purified drinking water; the NF choline supplemented (NFCS) group received the NF diet and purified drinking water supplemented with 25 mM of choline chloride; the high-fat control (HFCO) group received a high-fat (HF) diet (D12492, Research Diets) containing 60% kcal from fat and purified drinking water; and the HF choline supplemented (HFCS) group received the HF diet and purified drinking water supplemented with 25 mM of choline chloride (Supplementary Figure 1). Male mice for mating received the NFCO diet. We previously reported that total choline contents were 11.7 mmol/kg in the HF diet and 7.6 mmol/kg in the NF diet, and that the level of choline supplementation yielded 4.5 times total choline intake in the CS vs. control groups (CO) (15). This supplementation level in the maternal diet was consistent with what was used in previous studies to improve offspring cognitive development and placental functioning in several studies (15, 22–24).

After 4 weeks of feeding with experimental diets, female and male mice were timed-mated in a 2:1 ratio. If a vaginal plug was detected in the morning, the female mouse was separated from the male mouse and time was recorded as embryonic day 0.5. Female mice continued to receive their assigned diets during gestation. A cohort of mice was dissected at E17.5 to investigate how fetal tissue responded to dietary treatments in late gestation. The rest of the dams continued their assigned diets until birth of pups. Thereafter, all dams were provided with the NFCO diet during lactation until weaning of pups at postnatal day 21. After weaning, two male and two female offspring were randomly chosen from each litter and fed the HF diet *ad libitum* without CS for 6 weeks before dissection, except for the absolute control (ABS) group which received the NF diet without CS both before and after birth. The ABS group contained 6 offspring of each sex. The other groups contained 8 offspring per sex.

### Sample Collection

Mice were euthanized by carbon dioxide inhalation after 6-h fasting. For the E17.5 cohort, mice fetuses were separated from the uterus of the dam and the fetal liver and brain were isolated. For the postnatal cohort, the liver, gonadal fat, and brain hippocampus were dissected. The samples were rinsed in phosphate buffered saline and dried on absorbent paper immediately after isolation. The samples were then weighed on an analytical balance. Thereafter, they were flash frozen in liquid nitrogen and stored at −80°C, or fixed in 10% formalin, or immersed in RNAlater^®^ (Thermo Scientific, Grand Island, NY, USA) overnight before being stored at −80°C until analysis.

### Analytical Measurements

#### Histology

Liver and gonad fat samples fixed with 10% formalin were sent to HistoWiz Inc. (Brooklyn, NY) for further processing. The samples were embedded in paraffin, cut in 5 μm sections, and stained with H&E. Slides were then scanned from 1 × to 200 × magnification and converted to analyzable electronic files. The liver slides were examined for signs of steatosis and inflammation (25). Gonad fat samples were analyzed with Adiposoft in ImageJ to assess adipocyte size (26).

#### Global DNA Methylation

DNA was extracted from samples using the GeneJET Genomic DNA Purification Kit (Thermo Fisher, Suwanee, GA) following manufacturer's instructions. Thereafter, the isolated DNA was treated with nuclease P1, alkaline phosphatase, and phosphodiesterase (Sigma-Aldrich, St. Louis, MO) (27). CpG methylation of the samples was quantified with a DNA methylation ELISA kit (Cayman Chemical, Ann Arbor, Michigan) following manufacturer's instructions.

#### Site-Specific DNA Methylation

Genomic DNA (500 ng) was bisulfite treated using the EZ-DNA methylation kit (Zymo, Irvine, CA). DNA sequence of interest was amplified using PCR with the bi-sulfite treated DNA as the template and primers that incorporate the T7 tag (28). Sequences of interest were located in the promoter region of *Srebf1* and *Lep*, and differentially methylated region 0 (DMR0) of *Igf2* (29). Primer information was included in Supplementary Table 1. The amplicons were analyzed with the MassArray EpiTyper system (Agena Biosciences, San Diego, CA, USA) at the Epigenetic core facility of City University of New York (CUNY) Advanced Science Research Center (ASRC). This system uses matrix-assisted laser desorption ionization/time of flight mass spectrometry to assess methylation of each CpG unit within the amplicon. Each CpG unit may contain one or more adjacent CpG dinucleotides that cannot be resolved individually. The CpG sites and unit information of each sequence measured were provided in Supplementary Figure 2. Only CpG units with over 75% of success rate across samples were included in the final analysis.

#### RNA Extraction and Quantitative Real-Time PCR

RNA was extracted from one female and one male liver or gonadal fat sample from each litter using the TRIzol^®^ reagent (Thermo Scientific). Reverse transcription was conducted using the High-Capacity cDNA Reverse Transcription kit (Thermo Scientific) following the manufacturer's instructions. Gene transcript abundance was analyzed by quantitative real-time PCR with SYBR green detection using the CFX384 Touch™ Real-Time PCR Detection System (Bio-Rad, Hercules, CA, USA) as previously described (15). Data were expressed as the fold difference of the gene of interest relative to the housekeeping gene, beta-actin (*Actb*) using the 2^−ΔΔCt^ method (30). All primers were designed using GeneRunner Version 3.01 (31) (Supplementary Table 1).

#### SAM and S-Adenosylhomocysteine Measurements

SAM and SAH levels in liver samples were measured with a commercial ELISA kit (Cell Biolabs, San Diego, CA) following manufacturer's instructions.

#### Choline Metabolite Measurements

Whole fetal liver and brain samples, as well as 50 μg of postnatal liver samples ground in liquid nitrogen were used for choline extraction and quantification. Measurements were conducted using liquid chromatography- mass spectrometry (LC-MS)/MS methodology (32).

### Statistical Analyses

General linear models (GLMs) followed by *post-hoc* least significant difference (LSD) tests were constructed to assess the differences in the dependent variables among the dietary treatment groups. We also included sex as a fixed factor in the model and evaluated its interaction with dietary treatment. If the 2-way interactions had a *p*-value ≤ 0.1, we stratified the data by sex of the offspring. Dependent variables with residuals that deviate from the normal distribution were logarithmically transformed before analysis. A *p-*value < 0.05 was considered as significant. Values are presented as means ± standard error of mean (SEM).

## Results

### CS During Maternal HF Feeding Normalizes Offspring Adipocyte Size

**Figure 1:**
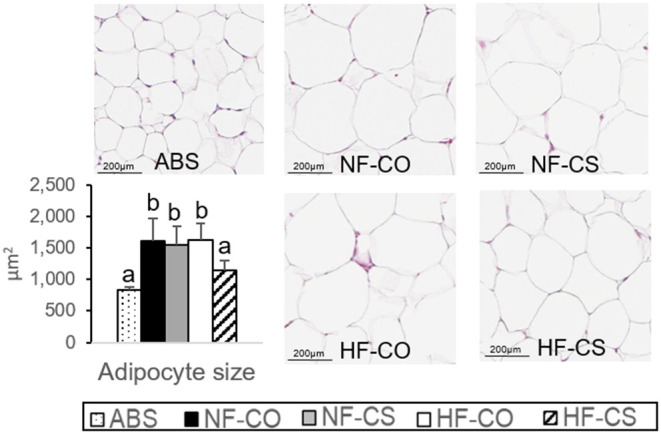
Normalization of adipocyte size by choline supplementation. H&E staining of offspring visceral adipose tissue after 6-week post-weaning high-fat feeding. Adipocyte size was assessed by the Adiposoft plugin in ImageJ. Different letters (e.g., a, b) represent statistically significant difference, *p* < 0.05. *n* = 6–8 per sex per group. ABS, absolute control; CO, control without choline supplement; CS, choline supplemented; HF, high-fat; NF, normal-fat.

We earlier described that maternal HF feeding led to higher fetal whole body adiposity and hepatic triglycerides while maternal CS mitigated these adverse alterations at E17.5 (16). In male offspring who received CS during prenatal HF feeding (i.e., the HFCS group), there was no difference in weight gain yet improved blood glucose control compared to the control group after 6-week post-weaning HF feeding (17). In this study, we examined liver histopathology after the 6-week post-weaning HF feeding, but did not find any sign of steatosis or inflammation in the samples from different groups (Supplementary Figure 3). Post-weaning HF feeding for 6 weeks increased VAT adipocyte size, yet this increase was prevented in the prenatal HFCS group (Figure 1), consistent with the lower leptin secretion implicated with CS compared to other post-weaning HF groups as we previously reported (17).

### Time-Specific Changes in Global DNA Methylation in the HFCS Offspring

**Figure 2:**
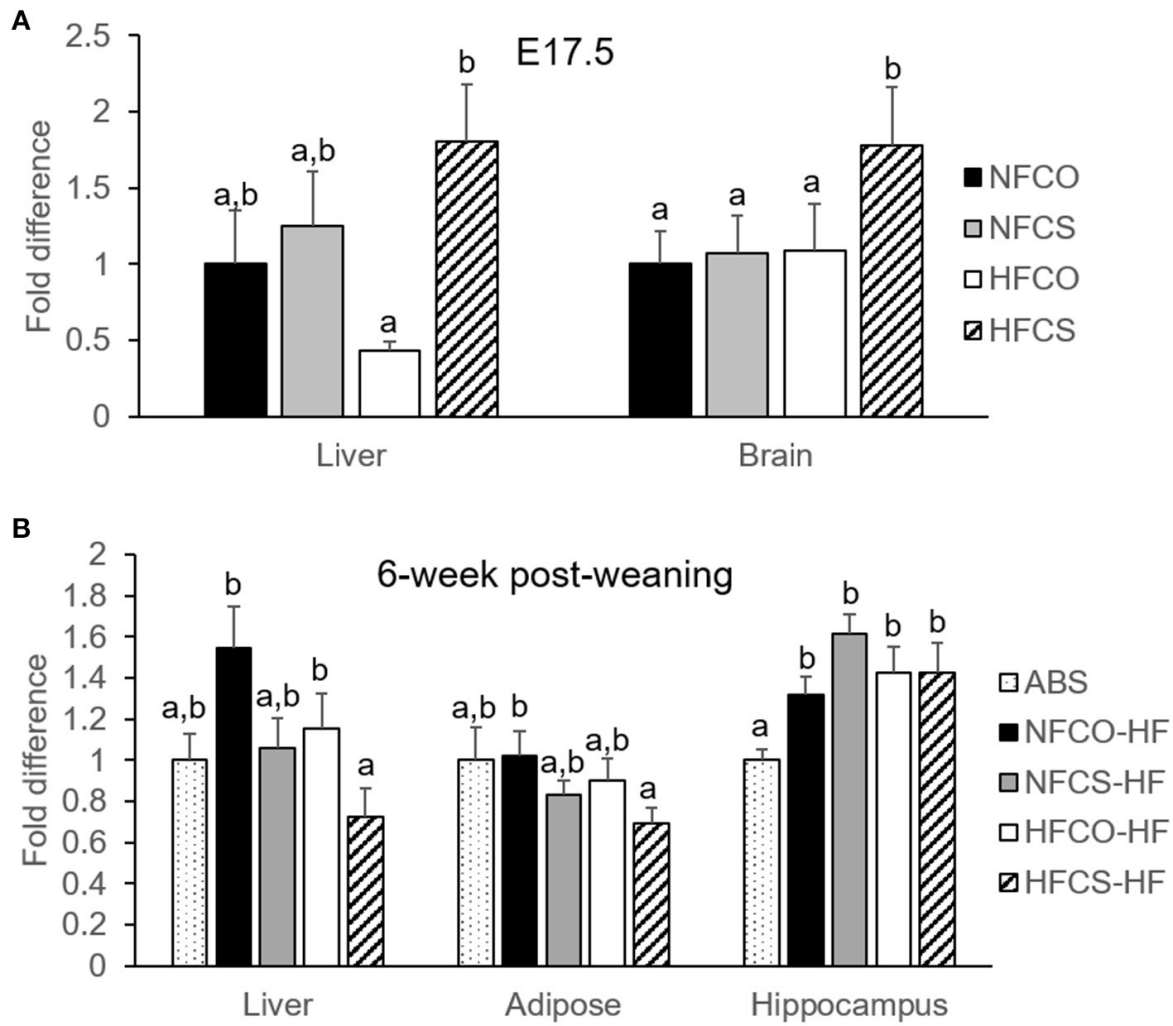
Maternal CS-mediated global DNA hypermethylation was eliminated under postnatal HF feeding. Global DNA methylation of mouse offspring with different maternal diets at E17.5 **(A)** and after 6-week post-weaning high-fat feeding **(B)**. Different letters (e.g., a, b) represent statistically significant difference, *p* < 0.05. *n* = 6–8 per sex per group. ABS, absolute control; CO, control without choline supplement; CS, choline supplemented; HF, high-fat; NF, normal-fat.

The lasting influence of maternal diet on offspring health is often imposed by an epigenetic mechanism. We therefore measured global DNA methylation of multiple tissues at prenatal (E17.5) as well as postnatal (after 6-week post-weaning HF feeding) periods. We observed significant increases in DNA methylation in the fetal liver in the HFCS group as compared to the HF, no choline supplementation control (HFCO) group (*p* = 0.002; Figure 2A). HFCS also increased DNA methylation in the fetal brain compared to all other groups (*p* < 0.05; Figure 2A). However, intriguingly the prenatal global methylation differences were not maintained at the postnatal time point. On the contrary, HFCS offspring had lower global DNA methylation in the liver than the HFCO and normal fat control (NFCO) offspring exposed to postnatal HF feeding (HFCS-HF vs. HFCO-HF and NFCO-HF, *p* < 0.05) (Figure 2B). DNA methylation of the VAT was also lower in the HFCS-HF vs. NFCO-HF offspring (*p* = 0.023; Figure 2B). In contrast, global DNA methylation in the postnatal brain hippocampus did not differ by prenatal HF or CS status. Even so, postnatal HF feeding increased global DNA methylation in the hippocampus compared to the ABS group (*p* < 0.01; Figure 2B).

### Maternal HFCS Increases Promoter *Srebf1* CpG Methylation During Both Pre- and Postnatal Periods

**Figure 3:**
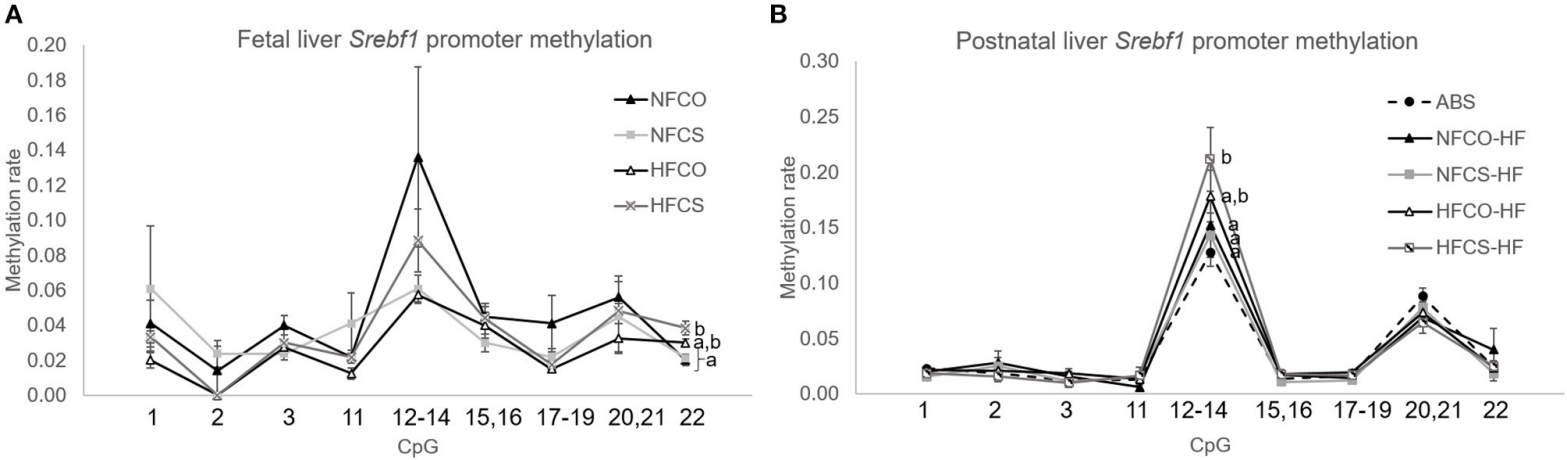
*Srebf1* promoter DNA methylation of mouse offspring was modified by maternal HF and CS. *Srebf1* methylation at E17.5 **(A)** and after 6-week post-weaning high-fat feeding **(B)**. Different letters (e.g., a, b) represent statistically significant difference, *p* < 0.05. *n* = 6–8 per sex per group. ABS, absolute control; CO, control without choline supplement; CS, choline supplemented; HF, high-fat; NF, normal-fat.

**Figure 4:**
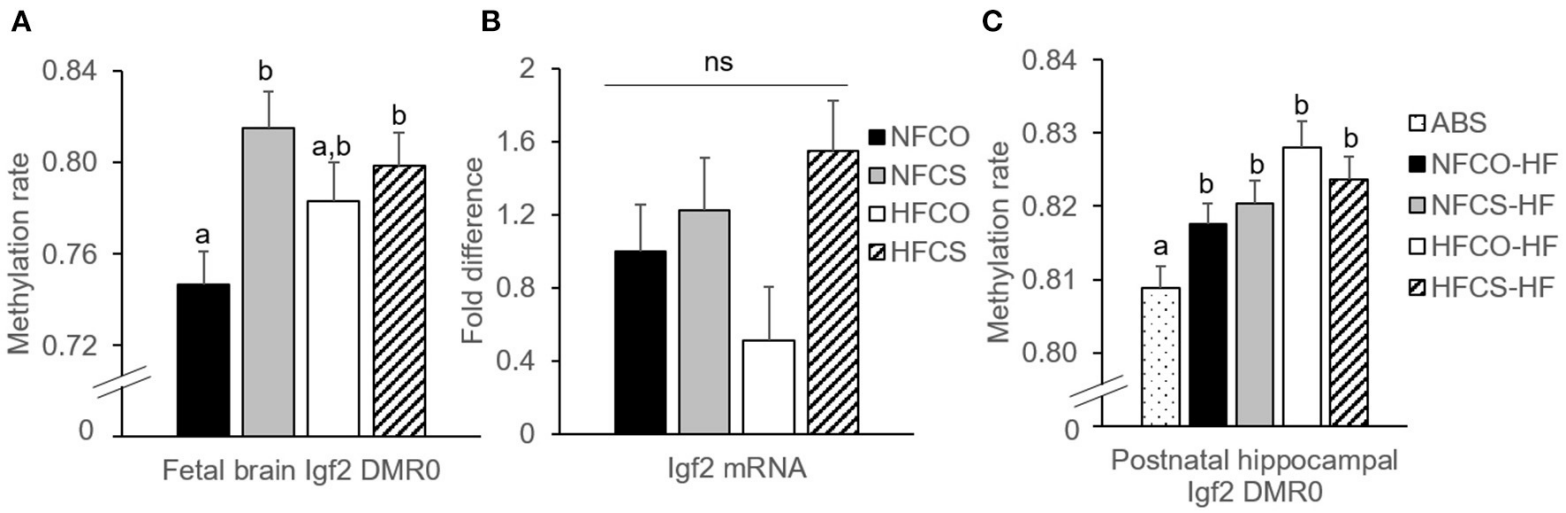
Brain *Igf2* differentially methylated region (DMR) 0 DNA methylation of mouse offspring with different maternal diets at E17.5 and after 6-week post-weaning high-fat feeding. **(A)** DMR0 methylation at E17.5. **(B)** *Igf2* mRNA expression at E17.5. **(C)** Hippocampal *Igf2* DMR0 methylation after post-weaning high-fat feeding. Different letters (e.g., a, b) represent statistically significant difference, *p* < 0.05; ns represents no significant difference. *n* = 6–8 per sex per group. ABS, absolute control; CO, control without choline supplement; CS, choline supplemented; HF, high-fat; NF, normal-fat.

Metabolic pathways involved with growth and macronutrient metabolism are especially susceptible to epigenetic programming by maternal exposures, thereby exerting lasting impacts on offspring cardiometabolic health (33, 34). We previously reported that mRNA expression of sterol regulatory element-binding factor 1 (*Srebf1*) that controls liver lipogenesis, was downregulated in the HFCS fetal liver, along with lower liver triglyceride accumulation in this group (16). We therefore measured site-specific methylation of *Srebf1* in the fetal liver. As expected, we found that at E17.5, methylation of one of the CpG units (containing CpG22 in the measured sequence) in the *Srebf1* promoter was upregulated by the combined effect of maternal HF and CS (HFCS vs. NFCS and NFCO, *p* < 0.01) (Figure 3A). This finding suggests that epigenetic regulation may be a mechanism by which CS influences *Srebf1* expression thereby affecting fetal hepatic lipogenesis during maternal HF feeding. We also explored DNA methylation marks in other growth and metabolism-related genes and found tissue- and gene- specific response to maternal HF and CS. Insulin-like growth factor 2 (*Igf2*) is a critical gene that promotes fetal growth. In contrast to *Srebf1*, maternal HF diet or CS did not alter *Igf2* differentially methylated region 0 (DMR0) methylation in fetal liver (Supplementary Figure 4A). In contrast, CS in both the HFCS and NFCS groups yielded higher methylation of *Igf2* DMR0 than the NFCO group in the fetal brain (*p* < 0.05; Figure 4A). Even so, this methylation pattern did not significantly affect *Igf2* mRNA expression in fetal brain, amongst all the groups (Figure 4B).

To understand whether these site-specific methylation changes were maintained with the postnatal obesogenic challenge, we measured them at post-weaning followed by 6-week of HF feeding. We observed significantly higher methylation of a CpG unit (containing CpG12-14) in the *Srebf1* promoter in the HFCS-HF group compared to the NFCS-HF, NFCO-HF, and ABS groups (*p* < 0.05; Figure 3B). However, the hypermethylation at CpG22 in the *Srebf1* promoter found in HFCS fetal livers (Figure 3A) was lost at this postnatal time point. Despite the higher methylation in CpG12-14 of *Srebf1*, the *Srebf1* mRNA expression was not altered amongst these groups, as reported previously for this postnatal time point (17). In contrast, CpG10 in the *Igf2* gene was hypermethylated in the NFCO-HF offspring than the other groups in the liver (*p* < 0.05) and, prenatal CS did not impose any effect on *Igf2* methylation (Supplementary Figure 4B). Like the global DNA methylation, we observed higher *Igf2* DMR0 methylation in all postnatal groups receiving a post-weaning HF diet compared to the ABS group in brain hippocampus (*p* = 0.002; Figure 4C). Prenatal HF feeding further increased methylation on several CpG units of *Igf2* DMR0 in the hippocampus. Specifically, both HFCO-HF and HFCS-HF had higher methylation (*p* < 0.05) in CpG1 and CpG3 than the ABS and NFCO-HF groups. Although lower leptin secretion accompanies CS, *Lep* methylation in VAT was unaltered amongst the various groups indicating a different mechanism for this gene regulation (Supplementary Figure 4C).

### SAM:SAH Ratio

The universal methyl donor SAM provides its methyl group for DNA methylation and gets converted to SAH afterwards. We therefore examined tissue SAM:SAH ratio, as this is often used as an indicator of methylation potential in the tissue (35). Neither SAM nor SAH concentrations were different among the groups in the fetal liver. Interestingly, the SAM:SAH ratio was significantly lower in the NFCS than the NFCO and HFCO fetal livers (*p* < 0.05; Table 1).

**Table 1:** SAM and SAH concentrations in fetal and postnatal livers.

	**NFCO**	**NFCS**	**HFCO**	**HFCS**	**ABS**	***p-*** **value**
**E17.5**						
SAM (mg/g tissue)	23.1 ± 3.7	13.1 ± 3.7	22.9 ± 3.7	22.4 ± 3.7		ns
SAH (mg/g tissue)	1.7 ± 0.8	2.5 ± 0.8	2.6 ± 0.8	3.0 ± 0.8		ns
SAM:SAH	15.1 ± 2.6^a^	5.9 ± 2.6^b^	13.7 ± 2.6^a^	9.6 ± 2.6^a,b^		ns
**6-week postnatal**						
**Male**						
SAM (mg/g tissue)	24.3 ± 2.1^b^	21.7 ± 2.3^b^	24.9 ± 3.3^b^	21.7 ± 3.5^b^	38.7 ± 7.7^a^	0.03
SAH (mg/g tissue)	0.91 ± 0.20	0.95 ± 0.14	0.79 ± 0.14	1.57 ± 0.73	1.03 ± 0.03	ns
SAM:SAH	30.8 ± 6.7	25.9 ± 7.0	36.8 ± 8.0	45.7 ± 26.9	37.2 ± 6.2	ns
**Female**						
SAM (mg/g tissue)	25.1 ± 4.6	23.5 ± 4.0	40.6 ± 26.1	44.5 ± 9.3	22.9 ± 2.2	ns
SAH (mg/g tissue)	0.52 ± 0.08	0.48 ± 0.03	0.79 ± 0.48	0.84 ± 0.16	0.60 ± 0.08	ns
SAM:SAH	48.0 ± 3.8	48.4 ± 7.0	48.4 ± 3.6	55.9 ± 8.4	56.5 ± 4.6	ns

*Different letters represent statistical significance at p < 0.05. ns, not significant (p > 0.05); n = 6–8 per sex per group. ABS, absolute control; CO, control without choline supplement; CS, choline supplemented; HF, high-fat; NF, normal-fat; SAM, S-adenosylmethionine; SAH, S-adenosylhomocysteine*.

However, during the postnatal period after 6-week HF feeding, the effects of NFCS on prenatal SAM and SAH were eliminated. Dietary treatments tended to interact with sex of the animals in determining SAM content (*p*_interaction_ = 0.1). After we stratified the analysis by sex of the animals, SAM content was lower in all postnatal HF groups than ABS (*p* < 0.05) in male offspring while female offspring had similar SAM status among all groups (Table 1). There were no differences in SAH or SAM:SAH ratio among the groups.

### Choline Metabolite Status

We previously reported that betaine levels were increased by maternal CS in the fetal liver at E17.5, while other choline metabolites were not altered (16). In this study, both maternal HF feeding and CS increased free choline levels in the fetal brain (*p* < 0.01). Therefore, the HFCS group had the highest levels of free choline in the fetal brain than the other groups (*p* < 0.01), while the NFCO group had the lowest level of free choline than the other groups (*p* < 0.01; Table 2). Other brain choline metabolites did not differ among the groups.

**Table 2:** Choline metabolite levels in fetal and postnatal tissues.

	**NFCO**	**NFCS**	**HFCO**	**HFCS**	**ABS**	***p-*** **value**
**Fetal brain**						
Methionine (nmol/g)	173 ± 10	215 ± 32	212 ± 26	196 ± 16		ns
Betaine (nmol/g)	0.53 ± 0.06	0.59 ± 0.09	0.52 ± 0.07	0.60 ± 0.06		ns
Choline (nmol/g)	60 ± 6^a^	110 ± 23^b^	114 ± 13^b^	162 ± 20^c^		0.006
GPC (nmol/g)	245 ± 11	199 ± 16	228 ± 11	218 ± 8		ns
Phosphocholine (nmol/g)	352 ± 28	339 ± 25	355 ± 24	339 ± 25		ns
Phosphatidylcholine (nmol/g)	1,748 ± 154	1,787 ± 226	2,001 ± 96	1,953 ± 194		ns
Sphingomyelin (μmol/g)	29.2 ± 0.5	30.0 ± 0.9	29.3 ± 0.3	28.9 ± 0.4		ns
**Postnatal liver**						
Methionine (nmol/g)	308 ± 58^b^	183 ± 23^a^	191 ± 20^a^	151 ± 11^a^	191 ± 25^a^	0.006
Betaine (nmol/g)	515 ± 73	637 ± 90	572 ± 76	607 ± 78	657 ± 85	ns
Choline (nmol/g)	309 ± 21	498 ± 65	382 ± 39	366 ± 36	408 ± 71	ns
GPC (nmol/g)	442 ± 49	465 ± 33	491 ± 30	447 ± 32	433 ± 32	ns
Phosphocholine (nmol/g)	190 ± 22^a^	454 ± 68^c^	367 ± 33^b,c^	364 ± 50^b,c^	301 ± 42^a,b^	0.008
Phosphatidylcholine (μmol/g)	20.5 ± 0.5^a^	22.9 ± 0.8^b^	21.3 ± 0.5^a,b^	21.2 ± 0.4^a,b^	22.9 ± 1.0^b^	0.05
Sphingomyelin (μmol/g)	1.07 ± 0.09	1.12 ± 0.09	1.08± 0.06	0.99 ± 0.06	1.14 ± 0.05	ns
Lysophosphatidylcholine (nmol/g)	297 ± 29	333 ± 13	191 ± 20	151 ± 11	191 ± 25	ns

*Different letters (a,b,c) represent statistical significance at p < 0.05. ns, not significant (p > 0.05); n = 6–8 per sex per group. ABS, absolute control; CO, control without choline supplement; CS, choline supplemented; HF, high-fat; NF, normal-fat*.

The 6-week post-weaning HF feeding significantly depleted methionine (*p* < 0.01) while increasing phosphocholine concentrations (*p* < 0.01) in the offspring liver (Table 2). Prenatal HFCS in the HFCS-HF group had no effect on methionine concentrations, but mitigated the increase in phosphocholine compared to the NFCO-HF offspring (*p* = 0.027). The HFCS-HF offspring also tended to have higher liver phosphatidylcholine (PC) concentrations than the HFCO-HF offspring (*p* = 0.05). The PC/phosphocholine ratio was decreased by postnatal HF feeding, demonstrated by the comparison between the NFCO-HF, HFCO-HF vs. the ABS group (*p* < 0.05). Prenatal HFCS in the HFCS-HF group completely abrogated the decrease in this ratio.

### DNMT Expression

**Figure 5:**
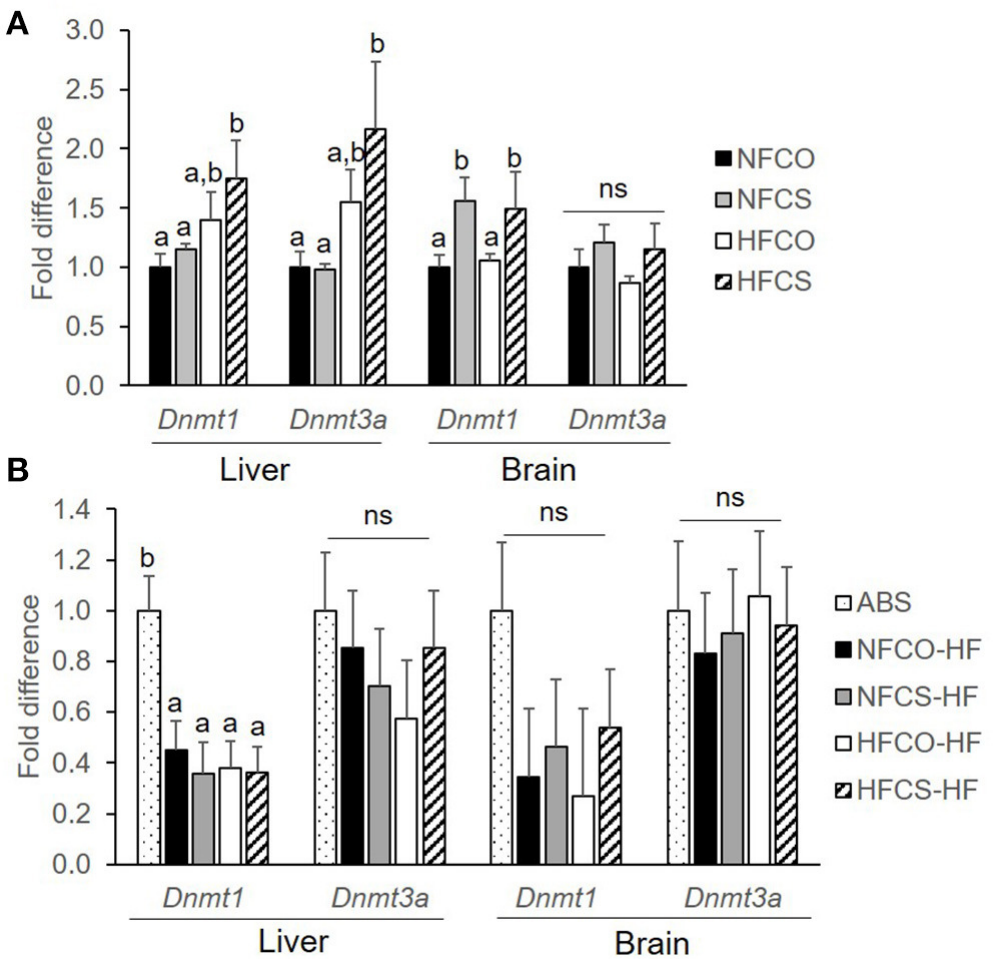
*Dnmt* mRNA expression of mouse offspring was upregulated by maternal CS in the prenatal period. *Dnmt* mRNA expression of mouse offspring with different maternal diets at E17.5 **(A)** and after 6-week post-weaning high-fat feeding **(B)**. Different letters (e.g., a, b) represent statistically significant difference, *p* < 0.05; ns, represents no significant difference. *n* = 6–8 per sex per group. ABS, absolute control; CO, control without choline supplement; CS, choline supplemented; HF, high-fat; NF, normal-fat. *Dnmt*, DNA methyltransferase.

Cellular DNA methylation is governed by expression of various DNMTs. Both *Dnmt3a* which mediates *de novo* methylation and *Dnmt1* which is responsible for methylation maintenance were upregulated in the fetal liver, at E17.5, in the HFCS group as compared to the NFCO and NFCS groups (*p* < 0.05; Figure 5A). Maternal CS during both NF and HF feeding led to upregulation of *Dnmt1* in the fetal brain as well (*p* = 0.015; Figure 5A).

The 6-week post-weaning HF feeding downregulated the expression of hepatic *Dnmt1* regardless of prenatal dietary exposures (*p* < 0.01). *Dnmt1* expression in the VAT and *Dnmt3a* expression in both the liver and VAT did not differ among the groups (Figure 5B).

## Discussion

Dietary choline has the potential to modify epigenetic programming of the offspring, thereby influencing metabolic health outcomes in the long term. In this study, we found that maternal CS during the prenatal period modified offspring DNA methylation in a time-specific manner and interacted with the HF feeding status of both the dams and the offspring.

Notably, maternal HFCS increased global DNA methylation in both offspring liver and brain in the fetal period, while paradoxically when faced with the postnatal HF environment, prenatal HFCS offspring demonstrated hypomethylation in both the liver and VAT. These findings corroborate previous contradictory studies that demonstrated the complex role of one carbon nutrient supplementation in epigenetic programming of offspring (2, 5–7, 35–41). For example, CS during pregnancy increased global DNA methylation of fetal livers in the tx-j mice (5) while gestational choline deficiency also led to higher DNA methylation of the fetal brain (8). Maternal one carbon nutrient intake or status in humans has been associated with higher, lower, or similar DNA methylation of genes such as *IGF2* in the children (39–41). Nonetheless, these studies suggest a more complicated relationship between one carbon nutrients and fetal programming rather than substrate provision.

Our data supports the model that the influence of maternal CS on offspring epigenetic marks might be time-specific and is subject to dynamic changes related to differential postnatal demands for choline. During the prenatal period when maternal CS is ongoing, the extra maternal supply of methyl groups provides an ample amount of methyl substrate for methylation reactions. In addition, the upregulation of DNMTs by maternal CS may further contribute to more active methylation reactions. Our results also corroborate similar findings in the toxic milk (tx-j) mouse model where maternal CS increased fetal liver global DNA methylation (5), and in a human controlled-feeding study when increased placental DNMT1 expression as well as global DNA methylation occurred with higher choline intake during the third trimester of pregnancy (7). Interestingly, although SAM:SAH is suggested as a marker of methylation potential (42), it was downregulated in the NFCS fetal livers. One possible explanation could be that more SAM was being consumed due to the more active DNMT activity. The inconsistency between the SAM:SAH ratio and tissue methylation status was observed in other studies as well. For example, Medici et al. reported that maternal CS had no effect on mouse fetal liver SAM or SAH although global DNA methylation was increased (5). In the aforementioned controlled-feeding human study higher choline intake in the third trimester increased placental global DNA methylation, yet did not alter SAM:SAH ratio (7). The disconnection between the SAM:SAH ratio and tissue methylation levels suggests that there is possibly a tight homeostatic mechanism in place and cautions us the use of SAM:SAH as a linear predictor of DNA methylation levels.

It is often viewed that DNA methylation marks established due to maternal one carbon supplementation can be maintained into later life, such as observations with the *agouti* mice where the transposable IAP promoter remained to be hypermethylated in the maternally one carbon supplemented offspring (2, 4, 43). However, whether difference in postnatal exposures may affect the stability of epigenetic marks established by prenatal one carbon supplementation has not been extensively explored. Some postnatal environment may increase or decrease the demand for methyl donors and conceivably has a direct impact on DNA methylation, and thus may eliminate the prenatally established epigenetic marks if methyl donors are not continuously supplemented during the postnatal life.

Previous studies have reported that maternal HF feeding increased DNA methylation in the offspring liver in one study yet decreased methylation in the offspring brain in another study (18, 19). Maternal HF feeding was also demonstrated to deplete one carbon metabolites including choline, betaine, and methionine in the offspring (18). However, Keleher et al. demonstrated that postnatal HF feeding overrode the epigenomic alterations elicited by maternal HF feeding (21). In this study, we demonstrated that indeed postnatal HF feeding is a strong modifier of brain DNA methylation, eliminating the higher global methylation that was marked by maternal CS during the fetal period. Moreover, when challenged by the postnatal HF environment, the prenatal HFCS group demonstrated lower global DNA methylation in both the liver and the VAT, contrary to the higher global methylation in this group during the fetal period. It is possible that the choline-rich environment during the prenatal period has increased these offspring's reliance on the dietary supply of choline for methyl group provision. Therefore, when faced with the postnatal HF environment, which may have depleted methyl donors as demonstrated by the lower methionine levels in offspring liver, the HFCS-HF group was not as capable of prioritizing choline use for methyl donation without continued CS. Moreover, there is a higher PC/phosphocholine ratio in the HFCS group, which may indicate more incorporation of choline into PC in this group under the stress of postnatal HF feeding. Flux analysis using isotope tracer will be needed to confirm this hypothesis, in the future. Unlike in the prenatal period when HFCS fetal livers had higher expression of DNMTs, DNMTs were not upregulated to overcome the DNA hypomethylation in the HFCS-HF offspring at the postnatal time point without continued CS. Taken together, a mismatch of abundant methyl nutrient supply during the prenatal period with limited methyl nutrient availability during the postnatal period may exacerbate the offspring's susceptibility to postnatal DNA hypomethylation when the demand for methyl groups is heightened by situations like HF feeding. Whether this global DNA hypomethylation would increase genomic instability and risk of hepatic diseases like steatohepatitis and hepatocellular carcinoma (44, 45) later in life requires further exploration.

Site-specific methylation of metabolic genes also demonstrated differences among the groups between life stages, though the alterations did not demonstrate a universal pattern across different loci that resembles the global DNA methylation changes. We observed increased methylations at CpGs in the *Srebf1* promoter in the HFCS liver, at the fetal and postnatal time points, although the specific CpG altered at each time point was not identical. The increased methylation of one of the CpGs at E17.5 was consistent with the lower expression of this gene in the HFCS fetal liver, providing a potential epigenetic mechanism by which maternal CS during HF feeding suppresses *de novo* lipid synthesis, thereby normalizing liver triglyceride and fetal adiposity that we reported in a previous study (16). Nevertheless, the increased methylation of this CpG was overridden by HF feeding at the postnatal time point. Instead, a different CpG had increased methylation in HFCS offspring, yet there was no corresponding difference in gene expression, suggesting that methylation at this site may not be solely essential for its regulation. *Igf2* is an important gene that regulates fetal and placental growth (46). It is an imprinted gene that contains multiple DMRs (29). The DMR0 that we measured had increased methylation in maternal CS fetal brains vs. controls, consistent with the role of choline for methyl group provision. Unexpectedly, gene expression levels did not differ accordingly. Since we did not measure other DMRs, it is unknown whether the consistent changes in other DMRs are needed to modify expression of this gene. Despite the lower global DNA methylation and smaller adipocyte size observed in VAT of the HFCS offspring, site-specific DNA methylation analysis of the leptin gene promoter did not find significant alterations, suggesting that genomic loci have different degrees of susceptibility to the maternal HF and CS treatments. A whole genome screening of methylome would have captured susceptible epigenetic marks more globally, a limitation in our study. Nevertheless, the selected key metabolic genes have provided unequivocal evidence that both HF and CS interact to modify site- and tissue-specific methylation in different developmental stages. This study may also inform future studies to examine the interaction between dietary methyl nutrients and other exposures, such as hormonal regulation, as previous studies have shown that steroid hormones regulate genome-wide epigenetic programming (47) and modify the activity of enzymes such as phosphatidylethanolamine N-methyltransferase (PEMT) involved in choline and other one carbon metabolism (48).

In conclusion, our study suggests that prenatal CS during maternal obesity (HF diet) interacts to impact DNA methylation in a tissue-specific manner in fetal mice, but these methylation marks are not fully maintained in offspring exposed to an obesogenic diet. While prenatal metabolism may benefit from DNA methylation marks established by prenatal CS, this may increase the susceptibility to hypomethylation in an obesogenic environment if choline is not continuously supplemented. The gene expression and functional implications of these time-specific methylation changes in response to the interaction of prenatal CS and HF require further exploration. This dynamic need of choline in early life may provide us with more insights about establishing and maintaining the epigenome.

## Data Availability Statement

The original contributions presented in the study are included in the article/Supplementary Material, further inquiries can be directed to the corresponding author/s.

## Ethics Statement

The animal study was reviewed and approved by Brooklyn College Institutional Animal Care and Use Committee.

## Author Contributions

HK and XJ designed the experiments and drafted the manuscript. HK, ST, JL, BD, KE, MD, SS, and SK conducted the experiments. ST, AS, JL, and JC edited the manuscript. All authors contributed to the article and approved the submitted version.

## Funding

This research was funded by the National Institute of General Medical Sciences (NIGMS) of the National Institutes of Health (NIH) (Grant Number: 5SC3GM132010).

## Conflict of Interest

The authors declare that the research was conducted in the absence of any commercial or financial relationships that could be construed as a potential conflict of interest.

## Publisher's Note

All claims expressed in this article are solely those of the authors and do not necessarily represent those of their affiliated organizations, or those of the publisher, the editors and the reviewers. Any product that may be evaluated in this article, or claim that may be made by its manufacturer, is not guaranteed or endorsed by the publisher.
