# Supplementary figures and images for: Maternal Choline Supplementation and High-Fat Feeding Interact to Influence DNA Methylation in Offspring in a Time-Specific Manner
Source: Front Nutr. 2022 Jan 28;9:841787. doi: 10.3389/fnut.2022.841787 (PMC8837519; doi:10.3389/fnut.2022.841787)

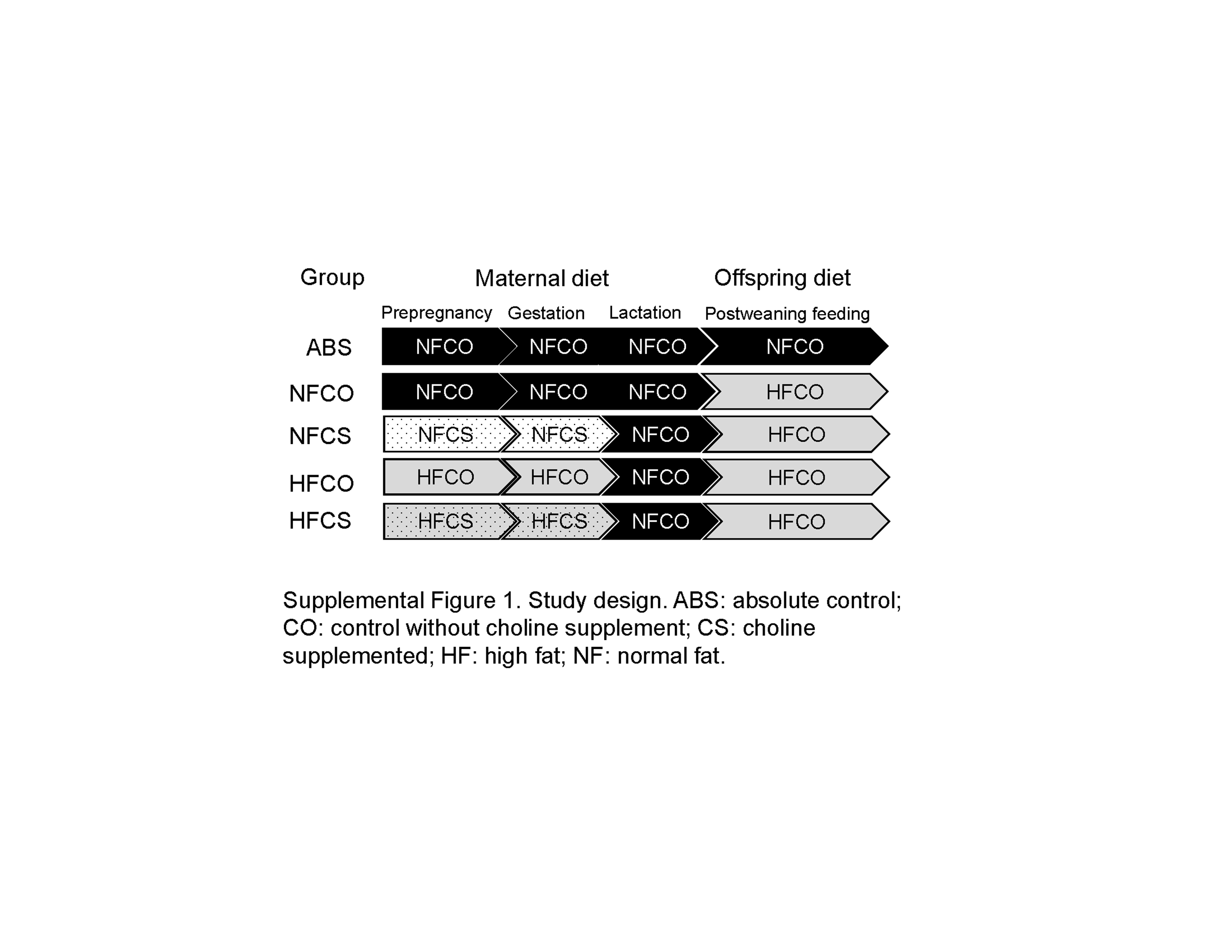

Supplement: Supplementary file 1 [file Image_1.TIF]

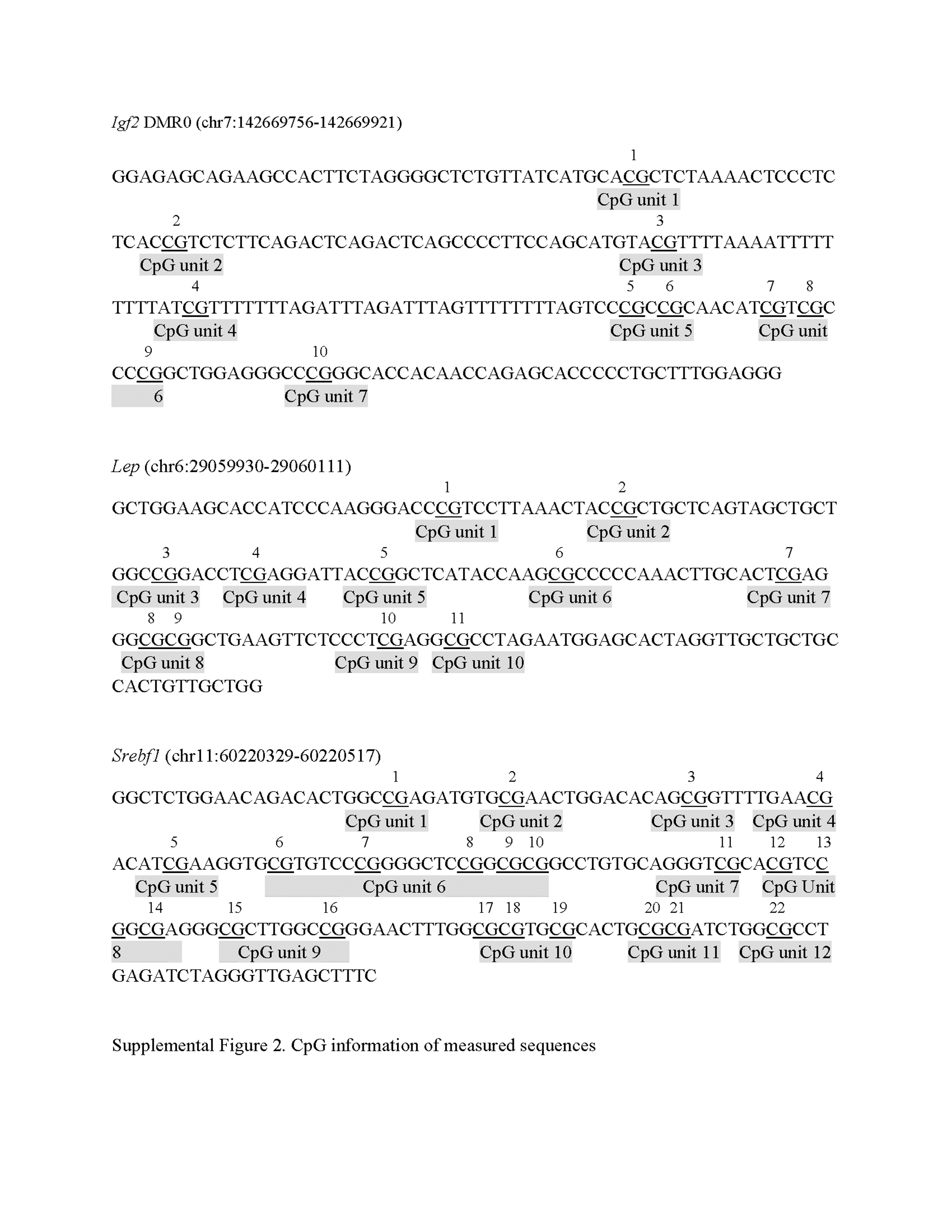

Supplement: Supplementary file 2 [file Image_2.TIF]

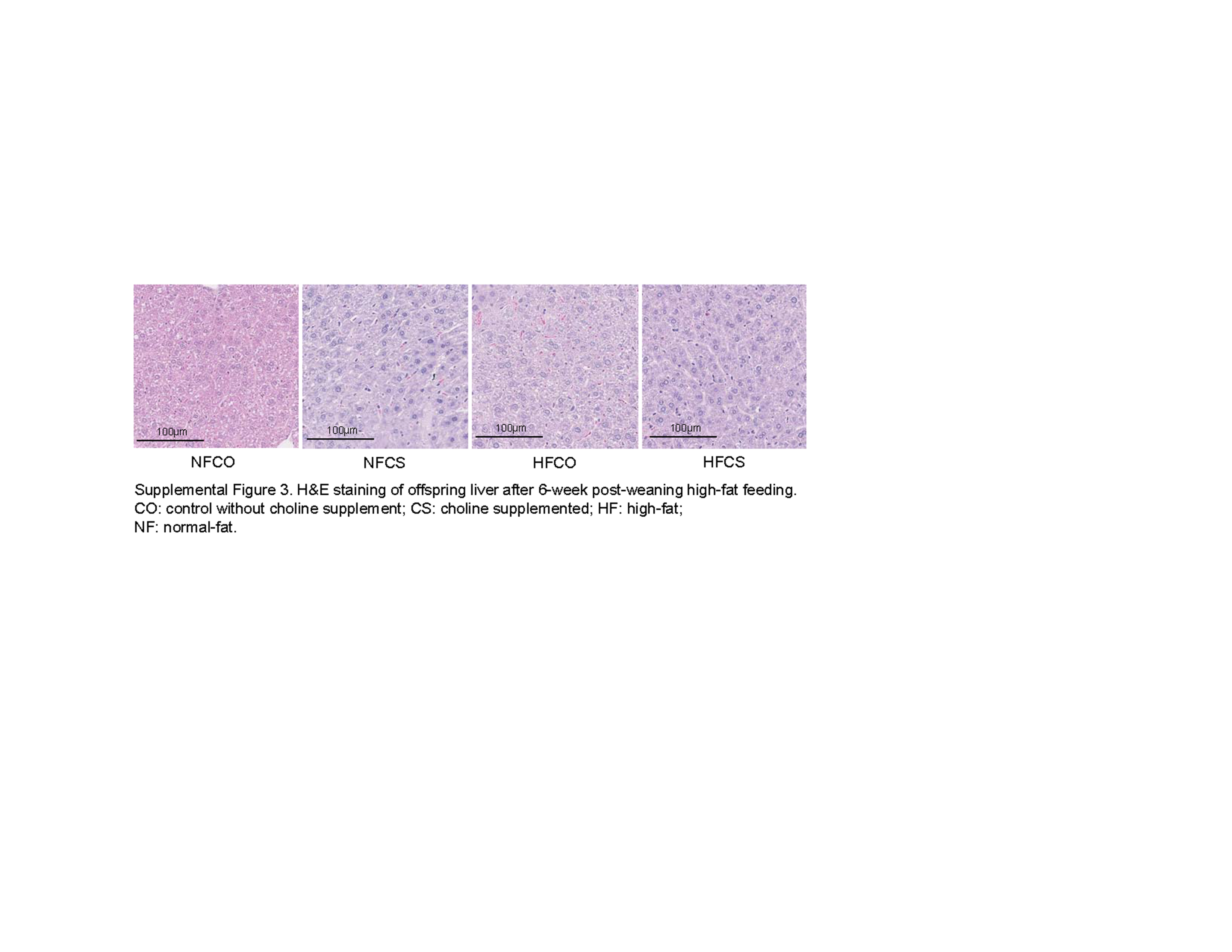

Supplement: Supplementary file 3 [file Image_3.TIF]

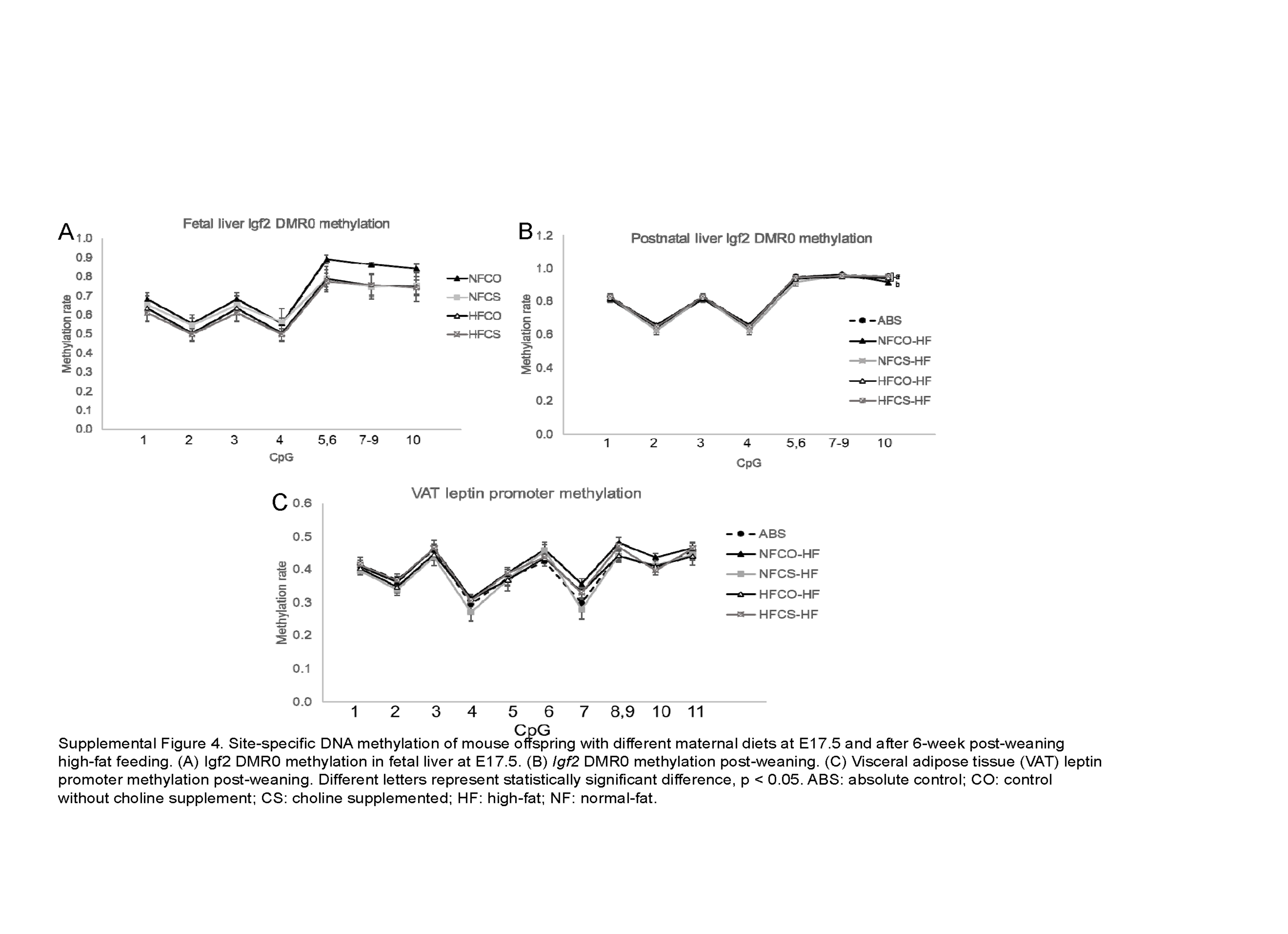

Supplement: Supplementary file 4 [file Image_4.TIF]
