# Supplementary material for: Maternal Choline Supplementation and High-Fat Feeding Interact to Influence DNA Methylation in Offspring in a Time-Specific Manner
Source: Front Nutr. 2022 Jan 28;9:841787. doi: 10.3389/fnut.2022.841787 (PMC8837519; doi:10.3389/fnut.2022.841787)
Supplement: Supplementary file 5 [file Table_1.DOCX]

Supplemental Table 1. Primers for site-specific methylation and mRNA expression analyses

|  | Forward primer | Reverse primer |
| --- | --- | --- |
| Site-specific methylation ^1^ |  |  |
| *Igf2* DMR0 | 5' GGAGAGTAGAAGTTATTTTTAGGGG 3' | 5' CCCTCCAAAACAAAAAATACTC 3' |
| *Srebf1* | 5' GGTTTTGGAATAGATATTGGT 3' | 5' AAAAACTCAACCCTAAATCTCAAAC 3' |
| *Lep* | 5' GTTGGAAGTATTATTTTAAGGGATT 3' | 5' ACCAACAACAATAACAACAACAAC 3' |
| mRNA expression |  |  |
| *Igf2* | 5’ GAGACATACTGTGCCACCCC 3’ | 5’ CGCGGACTGTCTCCAGGTGT 3’ |

^1^ The 5’ end of the forward primers for site-specific methylation analysis contains a 10-mer tag aggaagagag and the 5’ end of the reverse primers contains a T7 tag cagtaatacgactcactatagggagaaggct.
